# Comparative Analysis of Phenolic Composition of Six Commercially Available Chamomile (*Matricaria chamomilla* L.) Extracts: Potential Biological Implications

**DOI:** 10.3390/ijms221910601

**Published:** 2021-09-30

**Authors:** Maria Valeria Catani, Federica Rinaldi, Valentina Tullio, Valeria Gasperi, Isabella Savini

**Affiliations:** Department of Experimental Medicine, Tor Vergata University of Rome, 00133 Rome, Italy; nutrizionistarinaldi@gmail.com (F.R.); valentinatullio.nu@gmail.com (V.T.); savini@uniroma2.it (I.S.)

**Keywords:** chamomile, flavones, glutathione, oxidative stress, phytochemicals, polyphenols, reactive oxygen species

## Abstract

Several phytochemical-containing herbal extracts are increasingly marketed as health-promoting products. In particular, chamomile (*Matricaria recutita* L.) is well known for its anti-inflammatory, analgesic, and antitumor properties. Here, we evaluated differences in chemical composition among six commercially available products and their potential impact on biological activity in human immortalized colonocytes. Our investigation encompassed: (i) preparation of dry extracts and yield evaluation; (ii) qualitative and quantitative analysis of phenol content; (iii) modulation of redox state; and (iv) bioavailability of main bioactive compounds. We demonstrated that apparently identical products showed huge heterogeneity, in terms of yield extraction, chemical composition, and antioxidant effects. All samples contained high amounts of flavonoids and cinnamic acid derivatives, but differentially concentrated in the six extracts. Depending on polyphenol content, chamomile samples possessed variable antioxidant potential, in terms of decreased radical generation and increased reduced glutathione levels. The observed effects might be ascribed to flavones (apigenin, luteolin, and their glycones) highly represented in the six extracts. Nonetheless, chamomile extracts exerted cytotoxic effects at high concentrations, suggesting that a herbal medicine is not always safe. In conclusion, due to the complexity and variability of plant matrices, studies evaluating effectiveness of chamomile should always be accompanied by preliminary characterization of phytochemical composition.

## 1. Introduction

The term chamomile commonly refers to different daisy-like plants, belonging to the *Asteraceae* family, whose flowering tops are widely consumed as tisane. German chamomile (*Matricaria recutita* L.) is one of the best known species, native of the Mediterranean Basin and eastern Europe, and cultured worldwide. Infusions and essential oils from fresh or dried flower heads have aromatic, flavoring, and coloring properties, as well as positive health effects known since ancient times. The botanical name “*Matricaria*” comes, indeed, from the latin matrix (i.e., uterus), in reference to its relaxing ability for uterine muscles in menstrual and postpartum disorders. The anti-inflammatory and antioxidant activities of chamomile within the analgesic action are well known, so that this medicinal plant is usually used as liquid extracts, tablets, or capsules to decrease sleep and digestive tract disorders and to diminish anxiety and neuralgic pains (headache, sciatica, rheumatic pain) [1,2,3,4]. Topical application is useful in treatment of epidermis and mucous membrane inflammation (burns, sunburn, insect bites, psoriasis, eczema, and conjunctivitis) [5]. In addition to the aforementioned properties, recent studies have also shown a possible role of chamomile in the prevention of tumors, cardiovascular diseases, and diabetes [6,7].

The beneficial properties of chamomile are attributed to the presence of several bioactive compounds, which can generally be subdivided in two major classes on the basis of their solubility properties [4,8]. The hydrophobic group includes terpenoids (α-bisabolol and its oxidative derivatives) and azulenes (chamazulene), which are prevalent in the essential oil extracted from flowers and flower heads, and known for their anti-inflammatory, antiseptic, and antispasmodic properties [2,4,9,10,11,12,13]. The hydrophilic group comprises numerous polyphenols (mainly as flavonoid glycosides and lower amounts of free aglycones). Among them, the flavones apigenin, apigenin-7-O-glucoside, luteolin, and luteolin-7-O-glucoside are particularly abundant [4], although the flavonols quercetin and quercetin-3-O-rutinoside (also known as rutin) and the flavanone naringenin have also been found [8]. Chamomile is a source of other bioactive hydrophilic compounds, such as coumarins and some phenolic acids (including *p*-coumaric, caffeic, ferulic, and chlorogenic acids) [14,15,16]. It seems noteworthy that bioactive compound concentrations vary within different parts of the flower [17,18,19]; apigenin, for example, is highly concentrated in white ligulate flowers, while luteolin and quercetin are lower in ligulate flowers but are present at higher amounts in tubular flowers and in the receptacle [18]. It should also be underlined that polyphenols are plant-specialized secondary metabolites showing different roles in plant growth and development, as well as in response to biotic and abiotic stresses, such as nitrogen deficit, heavy metal accumulation, oxidative stress, temperature, UV lights, microorganism infestation, or herbivore attack [20,21,22]. Consequently, aside from variations depending on the anatomical part of the chamomile considered, significant differences in phytochemical levels are strongly due to extrinsic factors.

To date, these secondary compounds (and, in particular, flavonoids) have attracted the attention of scientists due to their ability to protect the human organism from oxidative stress, inflammation, and glucose metabolism dysregulation; these appear to be the most significant activities to prevent the onset of several chronic diseases, such as obesity, cardiovascular, and neurodegenerative diseases [6,7,23]. Of note, by acting through a broad range of molecular mechanisms encompassing all steps of carcinogenesis, the role of flavonoids as anticancer agents has been documented in cancer research [24,25,26]. For example, it has been reported that flavones can modulate cell growth signaling pathways, as in the case of luteolin-mediated induction of caspases and inhibition of cell cycle regulatory proteins CDK2 and cyclin D, as well as the apigenin-triggered downregulation of cyclin B1 and D1, Cdc-2, and Cdc-25 and upregulation of p53, p21, ERK, and p38 [27,28]. Given the high amounts of luteolin and apigenin, chamomile extracts have also been reported to exert antioxidant, anti-inflammatory, antiproliferative and proapoptotic effects in various human cancer cell lines [6,28,29]. Chamomile has been reported to significantly block IL-1β, IL-6 and TNFα-induced NO production and iNOS gene expression by inhibiting RelA/p65 activation [29] and to counteract H_2_O_2_-induced cellular damage by increasing activity of the transcription factor Nrf2, thus inducing the expression of several antioxidant enzymes, including NAD(P)H:quinone oxidoreductase, superoxide dismutase, and catalase [30]. Nonetheless, what emerges is that available current information on chamomile chemical composition is incomplete and often discordant, especially with regard to concentrations of individual phenolic compounds, thus confirming biological properties of different chamomile extracts might strictly depend on the phytochemical profile [31].

Based on this background, we compared ethanolic extracts of six commercially available chamomile products, determining chemical polyphenol composition and biological activity on human immortalized Caco2 colonocytes.

## 2. Results

### 2.1. Chamomile Samples Showed Significant Heterogeneity in Yield Extracts

**Figure 1:**
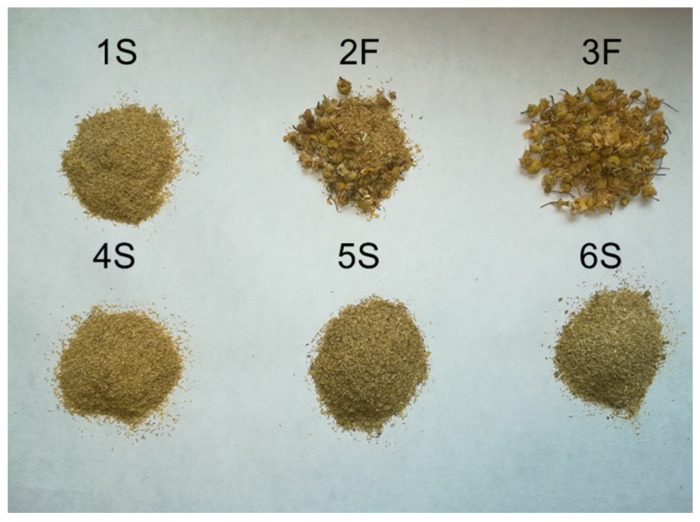
Image of the six different brands of chamomile samples used in the study.

To evaluate potential differences in the chemical composition of commercially available chamomile products, we selected six different brand chamomile samples, commonly sold in Italian supermarkets. As shown in Figure 1, four samples (1S, 4S, 5S, and 6S) exclusively contained the central part of sieved flowers, while in two samples (2F and 3F) all parts of the flowers (i.e., white ligulate and yellow tubular flowers) were present. Moreover, only the 3F sample derived from organic cultures.

We first analyzed extraction yields (expressed as milligrams of freeze-dried weight derived from 1 g of chamomile) of all samples. The ANOVA analysis of chamomile extracts showed significant heterogeneity (*p* < 0.0001) among samples: 3F > 2F > 6S = 5S = 4S > 1S. Amount of dry matter, indeed, was significantly higher in bloom samples (about 30% for 3F and 21% for 2F), while lower amounts were obtained for sieved chamomile samples (Table 1). All sieved extracts did not show significant differences, except for 1S sample showing the lowest yield (about 13%).

**Figure 2:**
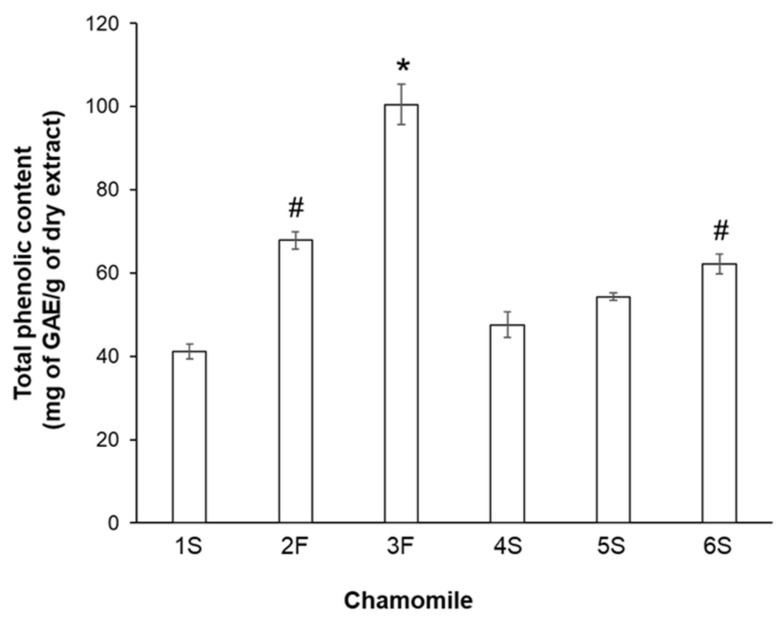
Total phenolic content in chamomile extracts. Reported values are expressed as milligrams of gallic acid equivalent (GAE) per gram of dry matter extract (mg of GAE/g dry extract). * *p* < 0.01 vs. all other samples; # *p* < 0.05 vs. 1S, 4S, and 5S.

As chamomile contains several biologically active compounds, in particular polyphenols (primarily flavonoids), we subsequently analyzed the phenolic profile of each sample. To this aim, we analyzed the total phenolic content by the Folin–Ciocalteu method. Statistically significant differences in phenolic content emerged among extracts, with the 3F sample showing the highest quantity of phenolic compounds (100.5 ± 4.8 mg of GAE/g dry matter) and 1S and 4S samples having the lowest content (41.1 ± 1.7 and 47.5 ± 3.1 mg of GAE/g dry extract, respectively) (Figure 2). Among sieved chamomile extracts, the 6S sample had the highest value. It seems noteworthy that the observed differences were not dependent on chamomile type (blooms or sieved), as similar phenolic amounts were displayed by bloom 2F chamomile and sieved 6S chamomile (67.9 ± 2.1 and 62.2 ± 2.3 mg of GAE/g dry extract, respectively). To evaluate whether such differences might be related to the lyophilization process, we also measured the total phenolic content in ethanol solutions, confirming that our extraction procedure did not lead to loss of phenolic components.

### 2.2. Chamomile Extracts Showed Significant Differences in Their Polyphenolic Profile

In order to achieve qualitative and quantitative determination of polyphenolic compounds, we performed HPLC analysis of the dry extracts. In particular, we focused on those compounds already described in the literature and known to be particularly abundant in chamomile organic extracts [32,33,34,35], such as hydroxycinnamic acids (chlorogenic, caffeic, *p*-coumaric, and ferulic acids), flavones (luteolin, apigenin, luteolin-7-O-glucoside, and apigenin-7-O-glucoside), and flavonols [quercetin (hydrate) and quercetin-3-O-rutinoside (rutin hydrate)]. We also attempted to evaluate the presence of other common flavonoids, not usually investigated in chamomile, such as the flavanones naringenin and diosmetin, the flavanols (±)-catechin (hydrate), (−)-epigallocatechin, (−)-epicatechin, and their gallate derivatives [(−)-catechin gallate, (−)-epicatechin gallate, and (−)-epigallocatechin gallate], and the isoflavone daidzein. HPLC chromatograms of each extract were characterized by the presence of about 30 main peaks; among them, a total of ten different polyphenolic compounds were identified by comparing retention times of peaks with those of known amounts of standards.

**Figure 3:**
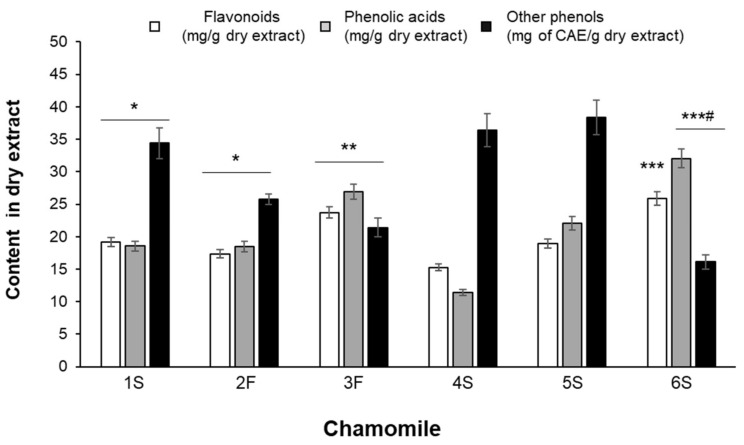
Total content of flavonoids, phenolic acids and other phenols identified by HPLC **analysis.** Results are expressed as mg of compound per gram of dry extract except for “other phenols” that are expressed as chlorogenic acid equivalents (CAE). Data are the means ± SEM of 3 independent extractions. * *p* < 0.05 vs. 4S and 5S; ** *p* < 0.01 vs. 1S, 2F, 4S and 5S; *** *p* < 0.001 vs. 1S, 2F, 4S and 5S; # *p* < 0.05 vs. 3F.

Although showing differences in their phenolic composition, all extracts were characterized by high content in phenolic acids and flavonoids (Figure 3). Statistically significant differences were found among samples: for example, the average content of hydroxycinnamics acids ranged from 11.41 ± 0.49 (4S sample) to 32.03 ± 1.43 (6S sample) mg/g of dry extract (Figure 3).

As detailed in Table 2, chlorogenic acid and *p*-coumaric acid were the predominant phenolic acids in all samples, with 3F and 6S samples showing the highest concentrations of chlorogenic acid and 5S and 6S samples being particularly rich in *p*-coumaric acid. Ferulic acid was also detected, especially in 3F and 5S samples. Caffeic acid was present at the lowest amounts in all extracts and its content did not statistically differ among samples.

Flavonoid total content was rather uneven among all samples, as it ranged from 15.27 ± 0.51 mg/g dry extract (4S sample) to 27.31 ± 0.51 mg/g dry extract (6S sample) (Figure 3). Each extract also differed in terms of specific flavonoids, although they contained mainly glycosides and fewer aglycons (Table 2). The highest content of apigenin-7-O-glucoside was found in the 3F and 6S samples, while the lowest was found in the 1S and 4S samples; luteolin-7-O-glucoside was highly abundant in the 6S sample, being slightly lower in all other extracts. In the 3F and 4S extracts, 4-fold higher apigenin levels than in the 1S sample were found; luteolin was particularly abundant in the 3F sample, showing a content 10 times higher than that detected in the 1S extract. The ratio between glycosides and aglycons ranged from 3 (4S sample) to 30 (6S sample) for apigenin, and from 3 (3F sample) to 24 (1S sample) for luteolin.

In this context it should be underlined that all the analyzed samples had a significant amount of apigenin-7-O-glucoside and this finding agreed with the European Pharmacopeia stating that chamomile flowers should contain at least 0.25% apigenin-7-O-glucoside [4]. The flavanol quercetin and its glycosylated form (rutin) were also present in chamomile samples, with the highest values found in 3F extract for the former, and in 1S and 4S samples for the latter. Concerning other flavonoids, no traces of naringenin, epichatechin, daidzein, catechin, or epigallocatechin gallate were detected (Table 2).

**Figure 4:**
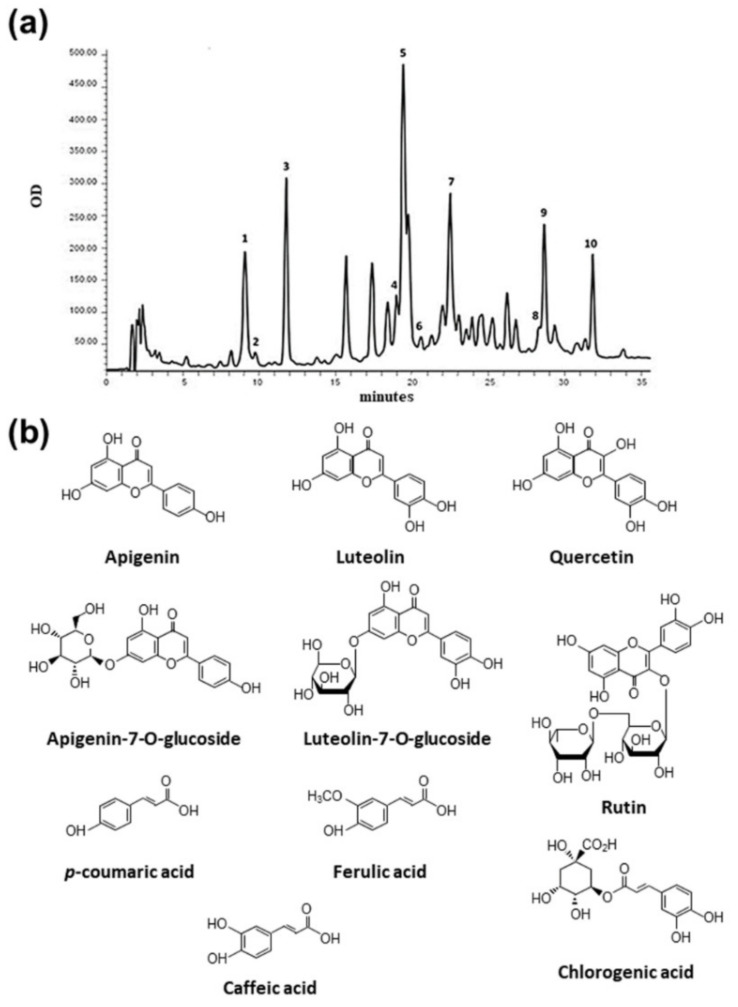
Chromatogram of 3F sample obtained with DAD detector set at 260 nm (**a**) and chemical structure of identified compounds (**b**). Identified peaks: 1. chlorogenic acid (9.07 min), 2. caffeic acid (9.74 min), 3. *p*-coumaric acid (11.77 min), 4. ferulic acid (19.01 min), 5. luteolin-7-O-glucoside (19.77 min), 6. rutin (20.60 min), 7. apigenin-7-O-glucoside (22.52 min), 8. quercetin (28.37 min), 9. luteolin (28.70 min), and 10. apigenin (31.89 min).

Heterogeneous amounts were also found for other phenolic compounds, corresponding to unidentified peaks, and considered of phenolic type based on their UV-vis spectra, characterized by absorption maxima between 260 and 330 nm. In particular, the 6S sample was found to be the poorest extract, showing 16.10 ± 0.85 CAE mg/g extract, while the 1S, 4S, and 5S samples showed the highest values (Figure 3). An example of a typical HPLC chromatogram, with the chemical structure of identified compounds, is given in Figure 4.

### 2.3. All Chamomile Extracts Dose-Dependently Affected Caco2 Cell Viability

Based on the above-observed quantitative differences in polyphenol profiles among the six chamomile products, we sought to investigate whether such differences might impact the in vitro biological activity of chamomile extracts.

The gastrointestinal tract is daily exposed to bioactive food components and is particularly responsive to the positive action of food components; therefore, we explored the efficaciousness of our extracts on human epithelial Caco2 cells. This cell line, originally derived from a colon carcinoma, is widely used as a model of the intestinal epithelial barrier, since it displays many properties typical of absorptive enterocytes [36].

**Figure 5:**
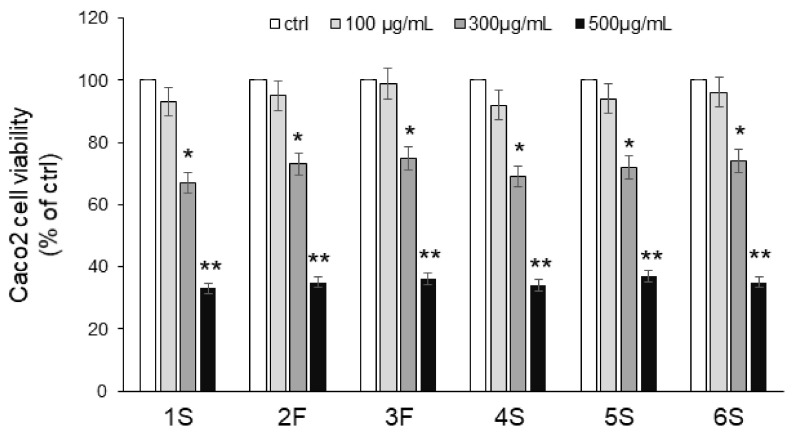
Effects of chamomile extracts on the viability of human epithelial Caco2 cells. Caco2 cells were incubated in the absence (ctrl) or in the presence of increasing concentrations (100–500 µg/mL) of chamomile extracts. After 24 h incubation, cells were harvested, and viability was assessed by MTT assay. Values are reported as percentage of control (ctrl) set to 100%. Data represent the mean ± SEM of at least three independent experiments, each performed in quintuplicate. * *p* < 0.05 and ** *p* < 0.001 vs. ctrl.

We first carried out an MTT assay to analyze the viability of Caco2 cells in response to 24 h incubation with increasing concentrations (0–500 µg/mL) of chamomile extracts. No extract exerted any effect on viability at the lowest concentration (100 μg/mL), while significant cytotoxic effects were observed with the highest concentrations: 300 μg/mL chamomile extract led to 30% inhibition, and 500 μg/mL reduced viability by about 70% (Figure 5). Based on these findings, we chose the lowest concentration for subsequent experiments. As apigenin and luteolin (as well as their corresponding O-glucoside derivatives) are the major constituents of chamomiles [32,33,34,35], deeply studied for their antioxidant activity [6,37], we also focused on these phytochemicals. We evaluated the effect of pure flavones at concentrations similar to those present in 100 μg/mL extracts, i.e., 0.5 µg/mL apigenin, 5 µg/mL apigenin-7-O-glucoside, 1.5 µg/mL luteolin, and 3 µg/mL luteolin-7-O-glucoside.

### 2.4. Chamomile Extracts Differentially Influenced Redox State of Caco2 Cells

**Figure 6:**
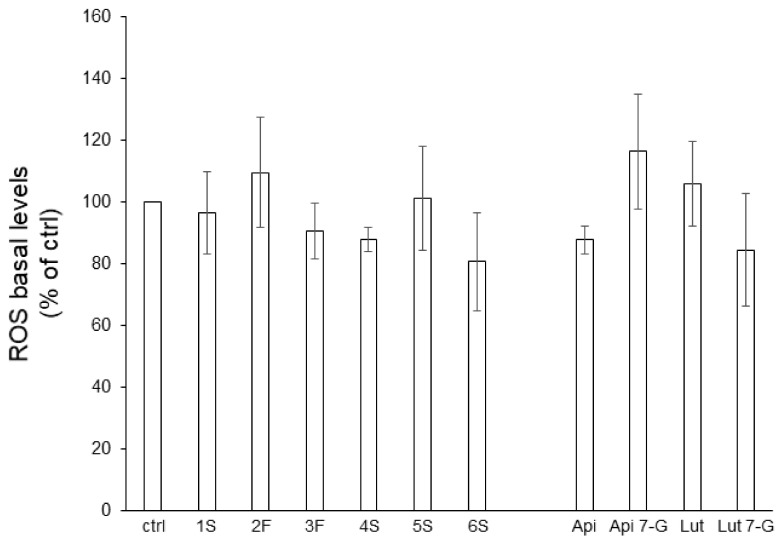
Effect of chamomile extracts and pure flavones on ROS basal levels. Caco2 cells were grown for 24 h in the absence (ctrl) or in the presence of 100 µg/mL of each extract or 0.5 µg/mL apigenin (Api), 5 µg/mL apigenin-7-O-glucoside (Api 7-G), 1.5 µg/mL luteolin (Lut), or 3 µg/mL luteolin-7-O-glucoside (Lut 7-G), and then incubated with 10 μM DCFH-DA for 30 min. Afterwards, ROS levels were quantified by FACS analysis. Data are reported as percentage of untreated cells (ctrl) arbitrarily set to 100%. Results represent the means ± SEM of 3 independent experiments, each performed in triplicate.

The anti-oxidant action of phytochemicals (as free-radical scavengers, oxidative stress relievers, and lipoperoxidation inhibitors) has deeply been investigated [29,30]. To this aim, we analyzed, by a cytofluorimetric approach, reactive oxygen species (ROS) generation in Caco2 cells pretreated for 24 h with chamomile extracts (or single flavones). Caco2 cells showed very low levels of endogenous ROS that neither extracts nor pure bioactive compounds significantly impacted (Figure 6).

**Figure 7:**
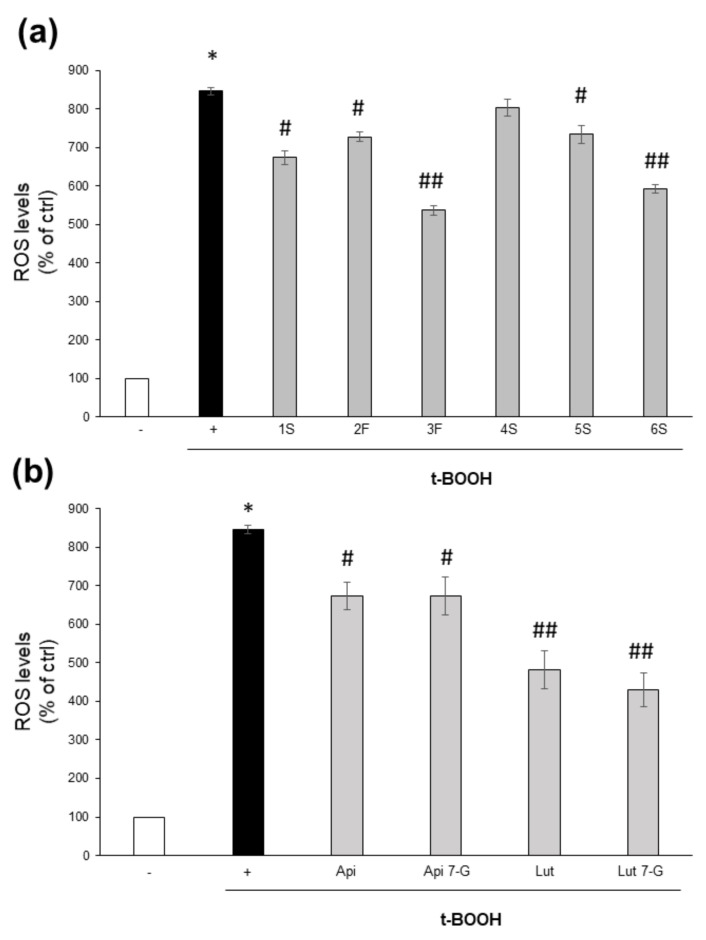
Effect of chamomile extracts and pure flavones on ROS levels. Caco2 cells were grown for 24 h in the absence (+) or in the presence of 100 µg/mL of each extract (**a**) or 0.5 µg/mL apigenin (Api), 5 µg/mL apigenin-7-O-glucoside (Api 7-G), 1.5 µg/mL luteolin (Lut), or 3 µg/mL luteolin-7-O-glucoside (Lut 7-G) (**b**), and then incubated with 10 μM DCFH-DA for 30 min. Afterwards, cells were left untreated (−) or treated with 2 mM t-BOOH; after 1 h incubation, ROS levels were quantified by FACS analysis. Data are reported as percentage of t-BOOH untreated cells (−) arbitrarily set to 100%. Results represent the means ± SEM of 3 independent experiments, each performed in triplicate. * *p* < 0.0001 vs. t-BOOH untreated cells (−) # *p* < 0.05 and ## *p* < 0.01 vs. t-BOOH-treated cells (+).

On the contrary, they were effective into counteracting the oxidative burst triggered by the potent pro-oxidant t-BOOH. Except for the 4S extract, all chamomile extracts were able to lower ROS increase triggered by 1 h treatment with t-BOOH; 3F and 6S samples were the most effective (Figure 7a). It is noteworthy that this effect was more evident for those extracts containing the highest total flavonoid content (Figure 4). Moreover, all tested pure compounds were significantly effective, with luteolin and luteolin-7-O-glucoside being the most efficacious; the two flavones reduced ROS levels to about 44% and 51%, respectively, with respect to t-BOOH-treated cells (Figure 7b).

**Figure 8:**
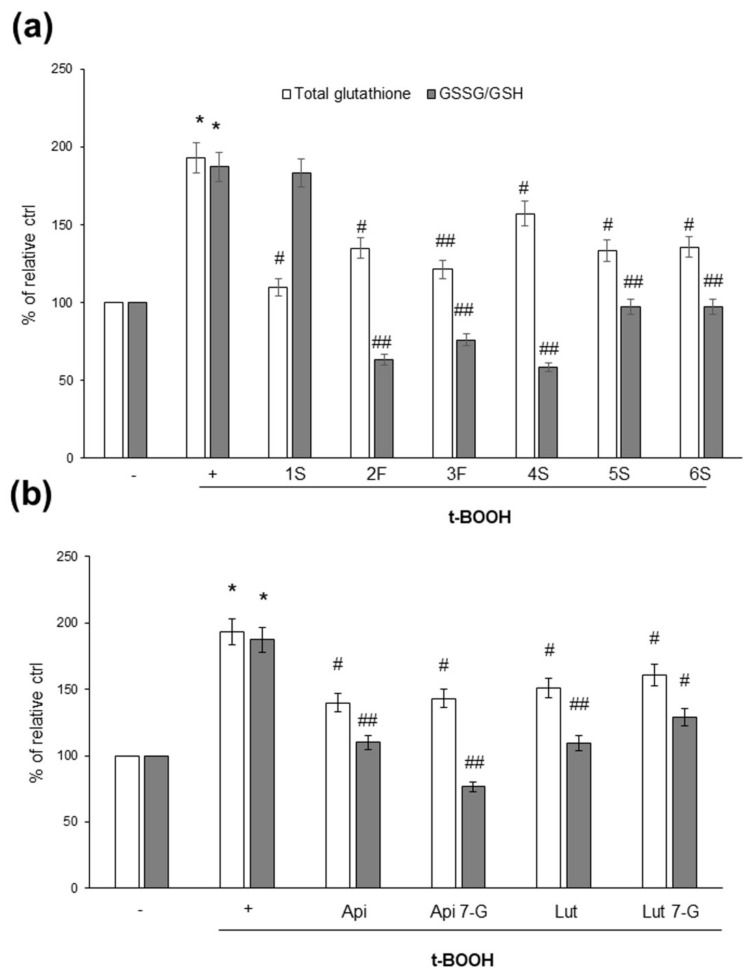
Effects of chamomile extracts and pure flavonoids on intracellular total glutathione content and ratio between the oxidized (GSSG) and reduced (GSH) form. Caco2 cells were grown for 24 h in the absence (+) or in the presence of 100 µg/mL of each extract (**a**) or 0.5 µg/mL apigenin (Api), 5 µg/mL apigenin-7-O-glucoside (Api 7-G), 1.5 µg/mL luteolin (Lut), or 3 µg/mL luteolin-7-O-glucoside (Lut 7-G) (**b**). Glutathione content was monitored in cells left untreated (−) or treated for 1 h with 2 mM t-BOOH, as described in Materials and Methods. Data are reported as percentage of t-BOOH untreated cells (−) arbitrarily set to 100%. Results represent the means ± SEM of 3 independent experiments, each performed in triplicate. * *p* < 0.0001 vs. t-BOOH untreated cells (−) # *p* < 0.05 and ## *p* < 0.01 vs. t-BOOH-treated cells (+).

As glutathione is the most important intracellular antioxidant, and its content and/or oxidation state reflects the redox balance within cells, we analyzed the effects of chamomile extracts (and phenolic compounds) on the intracellular content of total glutathione, as well as the ratio between reduced (GSH) and oxidized (GSSG) glutathione; the GSSG/GSH ratio is considered a very sensitive parameter of redox state perturbations [38,39]. As shown in Figure 8, t-BOOH increased total glutathione content, as well as the GSSG/GSH ratio. When we looked at the effects of the chamomile samples, we observed that not all plant extracts were able to impact the glutathione redox state; the 1S sample, indeed, appeared to be inefficacious (Figure 8a). Finally, all pure phenolic compounds were effective in modulating glutathione concentration, as well as the GSSG/GSH ratio (Figure 8b).

### 2.5. Luteolin and Luteolin-7-O-Glucoside Are Differentially Internalized by Caco2 Cells

**Figure 9:**
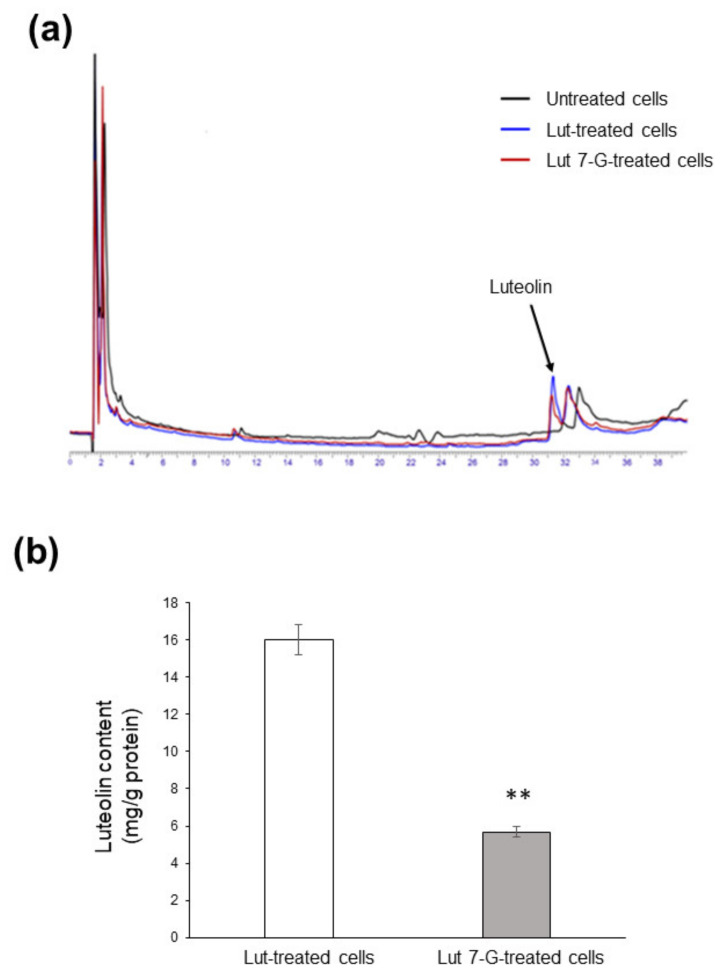
Uptake of luteolin and luteolin-7-O-glucoside by Caco2 cells. (**a**) Caco2 cells were left untreated (black line) or treated with 20 µg/mL of either luteolin (Lut-treated cells; blue line) or luteolin-7-O-glucoside (Lut 7-G-treated cells; red line) for 24 h, before HPLC analysis. (**b**) Concentration of luteolin inside cells as measured by HPLC. ** *p* < 0.01 vs. Lut-treated cells.

We finally evaluated flavonoid accumulation into Caco2 cells; we focused on luteolin and luteolin-7-O-glucoside because they proved to be the most active compounds. After 24 h incubation with either luteolin or luteolin-7-O-glucoside (both at 20 µg/mL), Caco2 cells transported both compounds across the plasma membrane. Luteolin was efficiently taken up, while luteolin-7-O-glucoside was not detectable inside cells, which instead contained only the corresponding aglycone (Figure 9a). This unexpected result may be explained by hypothesizing a rapid metabolism by cellular enzymes able to remove the sugar moiety. However, it should be underlined that uptake efficacy was different, since 3-fold lower flavone content was found in luteolin-7-O-glucoside-treated cells (5.67 ± 0.28 mg/mg proteins) with respect to luteolin-treated cells (16.03 ± 0.80 mg/mg proteins), indicating less bioavailability of the glycosylated form (Figure 9b).

## 3. Discussion

The present study evaluated differences in chemical composition and biological activity among six chamomile samples, commercially available in Italy. A significant heterogeneity, in terms of yield extraction, was found among samples; the 3F sample had the highest yield, while the 1S sample had the lowest. Average extraction values were comparable to those already reported in literature; for example, 21.7% yield of dry material from flower extracts of *Matricaria recutita L.* was reported following extraction with 70% aqueous ethanol [32] and 22.3% yield was observed after maceration in 50% aqueous ethanol [40]. Such differences did not depend on product type, since the 2F and 6S samples, which showed similar yield amounts, derived from intact whole (2F) and sieved central (6S) flowers. Moreover, differences did not even depend on the method we used for preparing dry extracts (70% ethanol extraction). Compared to other extraction techniques (such as Soxhlet procedure, steam distillation, and supercritical carbon dioxide), this well-documented and reproducible method provides the greatest yield and preserves the antoxidant activity of extracts; moreover, it also has the advantage of being a time- and labor-saving practice [4,32]

Differences in dry matter amounts were paralleled by dissimilarity in total polyphenol content; the 3F sample showed the highest amounts, while the 2F and 6S samples displayed comparable values (lower than 3F, but significantly higher than other samples). In agreement with the literature, our extracts mainly contained flavonoids (especially glycones) in copresence with cinnamic acid derivatives; ten phenolic compounds (apigenin, luteolin, apigenin-7-O-glucoside, luteolin-7-O-glucoside, quercetin, rutin, chlorogenic acid, ferulic acid, caffeic acid, and *p*-coumaric acid), differentially concentrated in the six extracts, were indeed identified. HPLC analysis also revealed that chamomiles commonly sold in Italy are not adulterated or substituted with close chamomile relatives; all analyzed chamomiles, indeed, met European Pharmacopeia’s requirements for dried capitula of *Matricaria recutita L.*, i.e., the total content of apigenin-7-O-glucoside has to be at least 0.25% [4].

Except for the 4S sample, all extracts were able to counteract ROS generation triggered by a pro-oxidant stimulus; however, chamomile extracts richer in flavonoids (especially flavones, such as apigenin, luteolin, and relative glycosides) and phenolic acids (namely, the 3F and 6S samples) possessed the highest antioxidant potential. In this context, it should be recalled that the Folin–Ciocalteau reagent is also reactive toward other compounds (such as proteins, carbohydrates, amino acids, nucleotides, thiols, unsaturated fatty acids, vitamins, aldehydes, and ketones), thereby being considered a measure of total antioxidant capacity rather than of phenolic content [41,42,43]; nonetheless, because phenolics are usually the most abundant antioxidants in plants, the Folin–Ciocalteau assay gives a rough approximation of total phenolic content.

From an economical point of view, what emerged from this analysis is that the potential beneficial effects of chamomile formulations are not linearly related to the cost of the product. Bloom samples were particularly expensive, with the organic 3F sample having the highest selling price (180.00 euro/Kg); on the contrary, the 6S sample deriving from conventional agriculture and showing the same chemical and biological properties as the 3F sample, as the cheapest chamomile (20.00 euro/Kg). Therefore, whether organic and/or more expensive products are the best in terms of bioactive compound enrichment remains an open question.

Dietary habits play a key role in maintaining a healthy state and the gastrointestinal tract, directly exposed to food, is notably responsive to dietary factors, which can exert either negative or positive effects in the development and prevention of intestinal malignancies [44,45,46]. In particular, oxidative (and nitrosative) unbalance is recognized as a crucial molecular mechanism underlying colorectal carcinogenesis [47,48,49,50,51] and several food components have been shown to promote intestinal epithelium transformation through a mechanism dependent on ROS (and/or reactive nitrogen species) generation. This is the case of heme-iron, nitrates and nitrites, present in red/processed meat [46,51,52,53], whose high consumption is associated with colorectal cancer risk [54,55]. Conversely, as emerged from clinical trials, systematic reviews, and metanalyses [54,56,57], dietary intervention with antioxidant compounds (vitamin C, selenium, vitamin E and β-carotene, magnesium, and folate) and foods (fruits and vegetables, fiber, and dairy products), alone or in combination, represents an attractive strategy for preventing and decreasing colon–rectal occurrence and progression.

By employing human epithelial Caco2 cells, a widely used model of the intestinal epithelial barrier [36], we demonstrated that, although with some variability, chamomile extracts might effectively protect gut from oxidative stress. Except for the 1S sample, all extracts were able to increase the content of reduced glutathione (GSH), fundamental for antioxidant defense, as well as for regulation of gene expression, signal transduction, cell proliferation, and apoptosis. It is, therefore, conceivable that a chamomile-dependent increase in GSH content is a means by which cells attempt to cope with the increase in ROS levels triggered by t-BOOH, thus maintaining an optimal redox status. This positive action might be ascribed to apigenin and/or luteolin (as well as to their glycones), which incidentally possess properties superimposable to those of chamomile (i.e., anti-inflammatory, anticarcinogenic, antispasmodic, and mild sedative properties) [6]. Indeed, the 3F sample was particularly enriched in apigenin and luteolin, whereas the 6S sample contained larger amounts of apigenin-7-O-glucoside and luteolin-7-O-glucoside; in line with this hypothesis, the 4S sample was the poorest in flavone content and the least efficacious in counteracting ROS generation. Noteworthy, the finding that luteolin-7-O-glucoside had effects superimposable to those observed for its corresponding aglycone, together with the evidence of its reduced bioavailability, suggest that the antioxidant effects might be attributed also to extracellular mechanisms. Flavonoids are able, indeed, to localize in the hydrophobic core of the lipid bilayer, as well as at the membrane interface, leading to alterations in membrane fluidity, thereby preventing interaction of free radicals with membrane lipids [58,59]. Therefore, either flavonoid chemical structures or their capability to perturb cell membrane homeostasis are both key factors impacting the nature and extent of their biological activity [60].

In conclusion, some particularly critical aspects concerning research on plant foods emerged from our investigation. First, plant extracts are complex food matrices containing different bioactive compounds, which can exert additive/synergic actions, thus making it difficult to identify which phytochemical is responsible for positive effects. Therefore, attention should be shifted not to single compounds, but instead to the whole chamomile matrix. Second, apparently identical products can have a different content in bioactive principles and, therefore, studies evaluating the effectiveness of herbal infusions or extracts should always be accompanied by a preliminary chemical characterization. Finally, the finding that high doses (300–500 µM) of chamomile extracts exerted cytotoxic effects on Caco2 cells strongly indicates that a herbal medicine, although “natural”, is not always better, healthier, and safer, maybe due to the presence of different, not always fully characterized, bioactive compounds. Hence, particular caution should be paid to overconsumption of natural drugs.

## 4. Materials and Methods

### 4.1. Materials

The purity of all standards was >98%. Gallic acid, hydroxycinnamic acids (chlorogenic, caffeic, *p*-coumaric, and ferulic acids), the flavones luteolin, apigenin, luteolin-7-O-glucoside, and apigenin-7-O-glucoside, the flavonols quercetin (hydrate) and quercetin-3-O-rutinoside (rutin hydrate), the flavanones naringenin and diosmetin, the flavanols (±)-catechin (hydrate), (−)-epigallocatechin, (−)-epicatechin, and their gallate derivatives [(−)-catechin gallate, (−)-epicatechin gallate and (−)-epigallocatechin gallate], and the isoflavone daidzein were from Cayman Chemical Co. (Ann Arbor, MI, USA). The flavone diosmetin was from Extrasynthese Co. (Lyon, France). Methanol Chromasolv for HPLC was from Sigma Chemical (St. Louis, MO, USA).

### 4.2. Chamomile Samples

Six chamomile samples, commonly sold in Italy, were used in the study (Table 3); four samples were sold as sieved chamomile and two as chamomile blooms. All samples came from traditional agriculture, except for one (3F) that derived from organic culture.

### 4.3. Chamomile Extract Preparation

Chamomile extracts were prepared from all samples, according to the procedure already reported [4,61,62]. Briefly, chamomile (1 gr each) was weighted and crushed with a marble pestle and mortar. Thereafter, powders were resuspended in 70% ethanol solution (5% *w*/*v*) and kept on a shaker at 4 °C in the dark. After 24 h, ethanol solutions were filtered through a series of Whatman filters (Whatman, Maidstone, UK) and, finally, passed through a 0.22 µm filter. Filtered extracts were concentrated under nitrogen efflux, frozen, and lyophilized. The resulting extracts were weighed before being dissolved in dimethyl sulfoxide (DMSO) (stock solution of 100 mg/mL), aliquoted, and stored at –80 °C until use. Each aliquot was used once.

### 4.4. Total Phenol Content Determination

Total phenolic content was determined through the Folin–Ciocalteau method, as previously reported [62,63]. Briefly, Folin–Ciocalteau reagent (Sigma Chemical, St. Louis, MO, USA) and chamomile extracts (100 mg/mL) were diluted, respectively, 10 and 200 times in deionized water; then, 25 μL of the diluted extracts were mixed with 75 μL of deionized water plus 200 μL of the diluted Folin–Ciocalteau reagent. After 5 min, 800 μL of 700 mM sodium carbonate solution were added and samples were incubated at 25 °C for 30 min. Thereafter, solution absorbance was determined at 765 nm (OD_765nm_) with the Lambda Bio 20 spectrophotometer (Perkin Elmer Analytical Instruments, Shelton, CT, USA); absorbance values were always within the linearity range of a calibration curve, drawn with increasing concentrations (0.5–20 μg/mL) of gallic acid. Total phenolic content was calculated by extrapolation from the standard curve and expressed as milligrams of gallic acid equivalent (GAE) per gram of dry matter extract (mg of GAE/g dry matter).

### 4.5. HPLC Analysis of Phenolic Compounds

Qualitative and quantitative analysis of phenolic compounds in chamomile extracts was determined by HPLC, as previously reported [62]. The chromatographic system was equipped with a spectrophotometric detector (Perkin Elmer Series 200 Photo Diode Array, Perkin Elmer Analytical Instruments, Shelton, CT, USA) system including, as analytical column, the Prodigy™ (Phenomenex Inc., Torrance, CA, USA) 5 µm ODS-2 150 Å, LC Column 1 (250 × 4.6 mm), plus SecurityGuard Cartridge C18 (4 × 3.0 mm) (Phenomenex Inc., Torrance, CA, USA). The elution program was a linear gradient from 100% solvent A [acidified water containing 5% *v*/*v* acetic acid:acetonitrile 85:15] to 100% solvent B [methanol containing 5% acetic acid] in 50 min and a 100% isocratic gradient of solution B in 10 min (flow rate of 1 mL/minute). Phenolic compounds were identified by comparison with retention times and UV spectra of relative standards and literature data [64]. Optimal separation of all investigated analytes was carried out in a total run time of 60 min. Unidentified peaks were considered to be of phenolic type on the basis of their UV-vis spectral characteristics, referred to as “other phenols” and expressed as chlorogenic acid equivalents (CAE).

### 4.6. Cell Culture and Treatments

Human colon Caco2 cells (ATCC, Manassas, VA, USA) were grown in Dulbecco′s Modified Eagle′s medium (DMEM) supplemented with 2 mM l-glutamine, 100 mg/L kanamycin, and 10% heat-inactivated fetal bovine serum (FBS) at 37 °C in a 5% CO_2_ humidified atmosphere. Depending on experimental procedures, subconfluent cells were incubated with chamomile extracts, apigenin, luteolin, apigenin-7-o-glucoside, or luteolin-7-o-glucoside, at the indicated concentrations and for the indicated time periods.

### 4.7. MTT Assay

Caco2 cells were seeded onto 96-well plate at a density of 1.5 × 10^4^ cells and incubated overnight. The next day, subconfluent cells were treated for 24 h at 37 °C with increasing concentrations of chamomile extracts (100–500 µg/mL). Cell viability was evaluated via 3-(4,5-dimethylthiazol-2-yl)-2,5-diphenyl tetrazolium bromide (MTT) assay, as reported [62]. Briefly, after treatments, the culture medium was removed, the MTT reagent (5 mg/mL) was added to each well, and cells were incubated at 37 °C for 3 h in the dark; formazan crystals were then dissolved in DMSO and color development was monitored at 590 nm in a multiwell scanning spectrophotometer (BS1000 Spectra count, Packard BioScience Co., Meridien, CT, USA).

### 4.8. Intracellular Reactive Oxygen Species (ROS) Measurement

ROS generation was assessed through flow cytometry, by using the membrane-permeable fluorescent probe 2′,7′-dichlorodiidrofluorescein diacetate (DCFH-DA; Molecular Probes Inc., Eugene, OR, USA). After treatments, cells were incubated with 10 μM DCFH-DA for 30 min at 37 °C in the dark. Then, cells were washed twice with PBS and incubated for further one hour with 2 mM tert-butyl hydroperoxide (t-BOOH). Cell cultures not treated with t-BOOH were used as controls [65]. Fluorescence was recorded at 530 nm by exciting at 488 nm, using an argon laser. Ten thousand events were evaluated for each sample and ROS levels determined by FlowJo software (BD, Franklin Lakes, NJ, USA).

### 4.9. Glutathione Content

Intracellular reduced (GSH) and oxidized (GSSG) glutathione content was quantified by 5,5′-dithio-bis (2-nitrobenzoic acid) (DTNB)-glutathione reductase recycling assay, as previously reported [66]. GSSG was selectively measured in samples where GSH was masked by pretreatment with 2-vinylpyridine. Values were expressed as nmol/mg proteins.

### 4.10. Flavonoid Intracellular Uptake

Caco2 cells (6 × 10^6^ cells/test) were incubated for 24 h with luteolin or luteolin-7-o-glucoside (both at 20 µg/mL). Then, cells were washed twice with PBS and incubated for 20 min with 2 mL of 70% methanol. After, the methanol solution was recovered, centrifuged at 15.000 × *g* for 10 min at 4 °C, and the resultant supernatant was dried and analyzed by HPLC, as described above. Flavonoids taken up by cells were expressed as mg of flavonoid/mg of total proteins.

### 4.11. Statistical Analysis

Data reported are the mean ± SEM of at least three independent experiments, each repeated at least in triplicate. Analysis of variance (ANOVA) followed by Bonferroni post-hoc comparisons were performed through the InStat3 program (GraphPad Software, San Diego, CA, USA) to evaluate differences among samples, except for flavone uptake experiments, where an unpaired *t*-test was used. Significant differences were accepted at a value of *p* < 0.05.

## Figures and Tables

**Table 1:** Yields of chamomile sample extraction.

Sample	Mg of Dry Matter/g of Chamomile	% of Yield
1S	127.76 ± 5.64	12.78 ± 0.56
2F	214.07 ± 5.11 ^*,#^	21.41 ± 0.51
3F	298.79 ± 5.61 ^**^	29.88 ± 0.56
4S	178.08 ± 5.58 ^*^	17.81 ± 0.56
5S	161.79 ± 7.75 ^#^	16.18 ± 0.78
6S	181.65 ± 9.28 ^*^	18.17 ± 0.93

* *p* < 0.001 2F vs. 1S and 5S; 4S vs. 1S; 6S vs. 1S; ** *p* < 0.0001 vs. all samples; # *p* < 0.05 2F vs. 4S and 6S; 5S vs. 1S.

**Table 2:** Phenolic profile of chamomile samples through HPLC analysis.

		1S	2F	3F	4S	5S	6S
	Compound	Content (mg/gr of Dry Extract)					
Flavonoids	Apigenin	0.25 ± 0.02	0.79 ± 0.05	1.22 ± 0.08 ^a^	1.10 ± 0.08 ^a^	0.58 ± 0.04	0.42 ± 0.03
	Apigenin-7-O-glucoside	4.46 ± 0.31	7.18 ± 0.5 ^b^	9.93 ± 0.69 ^a^	3.52 ± 0.24	7.45 ± 0.52 ^b^	11.69 ± 0.81 ^a^
	Luteolin	0.27 ± 0.02	1.18 ± 0.08	2.60 ± 0.18 ^a,c^	0.71 ± 0.05	1.02 ± 0.07	1.12 ± 0.08
	Luteolin-7-O-glucoside	6.39 ± 0.58	5.19 ± 0.36	6.51 ± 0.45 ^d^	4.70 ± 0.33	6.70 ± 0.46 ^d^	9.17 ± 0.64 ^a^
	Quercetin	1.39 ± 0.10	1.22 ± 0.08	1.97 ± 0.14 ^a^	1.07 ± 0.07	1.31 ± 0.10	1.47 ± 0.10
	Rutin	4.40 ± 0.30 ^a^	1.76 ± 0.21	1.58 ± 0.10	4.17 ± 0.29 ^a^	1.78 ± 0.12	2.01 ± 0.14
	Others	nd	nd	nd	nd	nd	nd
Phenolic *acids*	Chlorogenic acid	6.62 ± 0.46	6.09 ± 0.42	13.25 ± 0.92 ^a^	2.90 ± 0.20 ^a^	5.06 ± 0.35	14.10 ± 0.98 ^a^
	Caffeic acid	0.66 ± 0.05	0.52 ± 0.04	0.77 ± 0.05	0.51 ± 0.04	0.51 ± 0.04	0.72 ± 0.05
	Ferulic acid	2.79 ± 0.19	2.14 ± 0.15	2.85 ± 0.20 ^d^	1.88 ± 0.13	3.58 ± 0.25 ^a^	2.42 ± 0.17
	*p*-coumaric acid	8.46 ± 0.59 ^e^	9.70 ± 0.67 ^e^	10.05 ± 0.70 ^e^	6.11 ± 0.42	12.89 ± 0.89 ^a^	14.80 ± 1.03 ^a^

Data are the means ± SEM of 3 independent extractions. nd: not detected. ^a^ *p* < 0.05 vs. all other samples; ^b^ *p* < 0.05 vs. 1S and 4S; ^c^ *p* < 0.01 vs. 1S; ^d^ *p* < 0.05 vs. 2F and 4S; ^e^ *p* < 0.05 vs. 4S.

**Table 3:** Description of chamomile samples analyzed in the study.

Sample	Chamomile Type	Culture	Cost (€/kg)
1S	Sieved	Conventional	62.50
2F	Blooms	Conventional	106.78
3F	Blooms	Organic	179.36
4S	Sieved	Conventional	50.50
5S	Sieved	Conventional	60.00
6S	Sieved	Conventional	20.00

## Data Availability

Not applicable.
